# FGF23: A Review of Its Role in Mineral Metabolism and Renal and Cardiovascular Disease

**DOI:** 10.1155/2021/8821292

**Published:** 2021-05-17

**Authors:** Anna Kurpas, Karolina Supeł, Karolina Idzikowska, Marzenna Zielińska

**Affiliations:** Interventional Cardiology Clinic, Department of Interventional Cardiology and Electrocardiology, Central Clinical Hospital, Pomorska 251, 92-213 Lodz, Poland

## Abstract

FGF23 is a hormone secreted mainly by osteocytes and osteoblasts in bone. Its pivotal role concerns the maintenance of mineral ion homeostasis. It has been confirmed that phosphate and vitamin D metabolisms are related to the effect of FGF23 and its excess or deficiency leads to various hereditary diseases. Multiple studies have shown that FGF23 level increases in the very early stages of chronic kidney disease (CKD), and its concentration may also be highly associated with cardiac complications. The present review is limited to some of the most important aspects of calcium and phosphate metabolism. It discusses the role of FGF23, which is considered an early and sensitive marker for CKD-related bone disease but also as a novel and potent cardiovascular risk factor. Furthermore, this review gives particular attention to the reliability of FGF23 measurement and various confounding factors that may impact on the clinical utility of FGF23. Finally, this review elaborates on the clinical usefulness of FGF23 and evaluates whether FGF23 may be considered a therapeutic target.

## 1. Introduction

Fibroblast growth factors (FGF) are polypeptide growth factors with diverse biological activities [1]. They are involved in the following processes: angiogenesis, mitogenesis, cellular differentiation, cell migration, and repair of tissue damage. The FGF family consists of 22 members divided into seven phylogenetic groups.

Fibroblast growth factor 23 (FGF23) belongs, along with fibroblast growth factor 19 and fibroblast growth factor 21 to the endocrine subfamily and is the youngest member of a diverse FGF family of proteins. In the 20th century, Itoh et al. isolated a mouse cDNA and encoded FGF23, which was identified in the ventrolateral thalamic nucleus [2]. FGF23 is a 32 kDa polypeptide with an N-terminal and a C-terminal region with 251 amino acids [1]. It is a phosphaturic hormone secreted mainly by osteocytes and osteoblasts in bone but also expressed by salivary glands, stomach, and, in much lower concentrations, by other tissues, including skeletal muscles, brain, mammary gland, liver, and heart [3, 4]. FGF23 is a circulating endocrine hormone that plays a regulating role in mineral metabolism. Increases in FGF23 concentrations occur as a physiological response to maintain normal serum phosphate levels by inducing renal phosphate excretion.

Understanding the interaction of organ systems, primarily the skeleton, intestine, and kidneys, is crucial for managing the anomalies in calcium and phosphate metabolism. Calcium and phosphate balance is regulated by a number of hormones, including parathyroid hormone (PTH), vitamin D, and FGF23. However, a newly discovered FGF23 has become a part of the bone-kidney axis that influences vitamin D metabolism; it is also considered a phosphate regulating hormone.

Various clinical studies have already shown that elevated FGF23 levels are strongly associated with total and cardiovascular (CV) mortality, especially in patients with chronic kidney disease (CKD) [5–7]. This review will focus on the role of FGF23, which is considered an early marker of CKD and a potent CV risk factor. Furthermore, it will elaborate on the reliability of FGF23 measurement and the potential use of FGF23 as a target for therapy.

## 2. Mechanisms of Action of FGF23

The expression of FGF23 is regulated by phosphate and 1,25-dihydroxyvitamin D [1,25(OH)_2_D]. The latter stimulates the production of FGF23 and creates a negative feedback loop that regulates the production itself. There are also many other regulators of FGF23, e.g., bone mineralization and remodeling, phosphate, calcium, leptin, estrogen, glucocorticoids, and iron metabolism [8].

The members of the FGF family bind to FGF receptors (FGFRs); however, the affinity of FGF23 to FGFRs is weak [1]. FGF23 can only act in the presence of an alternative cofactor called *α*-Klotho, which mediates its binding to the receptor. Kuro-o et al. identified *α*-Klotho in 1997 as a novel gene that delays the aging process [9]. Any abnormality in the Klotho gene expression leads to infertility, arteriosclerosis, skin atrophy, or short life span. This implies that Klotho plays a crucial role in the effect of FGF23.

**Figure 1:**
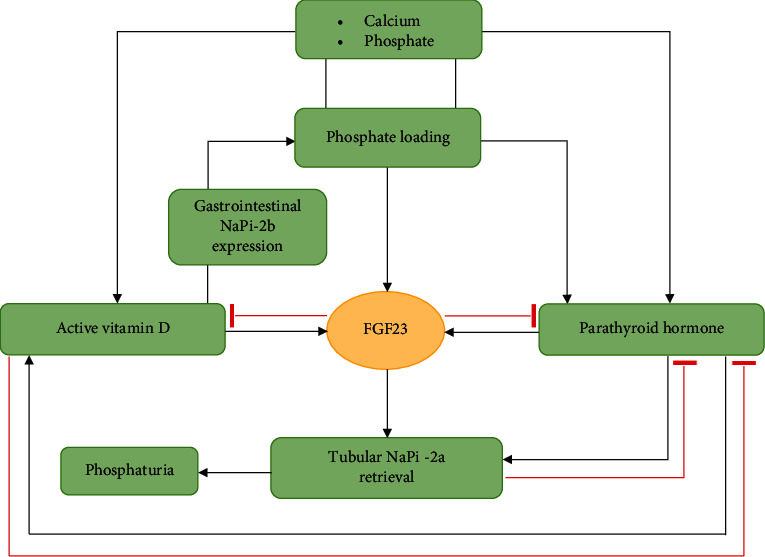
Physiology of FGF23, FGF23: fibroblast growth factor 23.

The FGF-receptor-*α*-Klotho complex in the distal renal tubule is the primary target for FGF23. In the kidney, FGF23 inhibits the production of the sodium phosphate cotransporters Npt2a and Npt2c, which increases phosphate reabsorption in the proximal renal tubule (phosphating effect) [10]. In addition, FGF23 suppresses the expression of 1-*α*-hydrolase, resulting in a decrease in active vitamin D formation. FGF23 inhibits also *α*-Klotho gene transcription by the kidney and the production of PTH in the parathyroid gland (see Figure 1). According to some medical reports, the PTH regulation of FGF23 is controversial [11–14].

The proteolytic processing of FGF23 is of fundamental importance for the regulation of its biological activity. A furin proprotein convertase cleaves an intact FGF23 between Arg179 and Ser180 into bioinactive N- and C-terminal fragments that do not reduce phosphate levels. Therefore, only full-length FGF23 shows biological activity [15].

## 3. Measurement of FGF23

There are two main types of commercial enzyme-linked immunosorbent assay (ELISA) that can be used for the determination of FGF23 in human plasma or serum: intact FGF23 (iFGF23) tests from Kainos, Immutopics (1^st^ and 2^nd^ generation), Millipore or DiaSorin, and C-terminal FGF23 (cFGF23) assay from Immutopics [16, 17]. The first detects only full-length FGF23 by simultaneous recognition of epitopes on the N- and C-terminal domains close to the proteolytic cleavage site, while the second detects both iFGF23 and C-terminal fragments by two antibodies against two epitopes within the C-terminal portion. Additionally, Shimizu et al. have invented an automated chemiluminescent iFGF23 assay that is superior to the widely used ELISA-based kits [18]. This methodology requires smaller samples and has a larger analytical range and shorter run times. However, none of the above assays has been validated for clinical use but for research purposes only.

All the assays vary considerably from each other. They use different antibodies targeting distinct epitopes on the FGF23 protein. The assays from Kainos and Immutopics are calibrated using recombinant human FGF23 expressed in murine myeloma cell lines. iFGF23 assays are calibrated in units of mass concentration-picograms per millilitre (pg/ml), while cFGF23 is calibrated in relative units per millilitre (RU/ml). Even the absolute values between the assays differ substantially as a result of different calibration and no previous harmonization of the tests. A commercial source of purified, native human FGF23 or international reference preparation of synthetic FGF23 is still missing.

### 3.1. Stability of FGF23

There is a controversial issue related to the requirements of samples for FGF23 tests because intact FGF23 may be degraded by proteases or modified after the blood withdrawal. Immutopics recommends the use of ethylenediaminetetraacetic acid (EDTA) anticoagulated plasma, while Kainos recommends the use of serum. Nevertheless, such a divergence is not clear; perhaps, it is related to the relative epitope stability in distinct sample matrices. Fassbender et al. presented conflicting data and suggested that plasma should be used in all FGF23 measurements [19]. Coherent results were obtained by Smith and colleagues (unpublished data) who showed that intact FGF23 is significantly more stable in plasma (with either lithium heparin or K_2_-EDTA anticoagulant) than in serum [20]. Australian researchers examined the effects of different specimen collection methods using plain (serum), EDTA (plasma), and EDTA with the addition of a protease inhibitor. They demonstrated that FGF23 levels measured in serum were remarkably lower as compared to plasma, and the addition of a protease inhibitor did not enhance the stability of FGF23. Although this issue has not been fully clarified, the researchers recommend plasma as the optimal collection method for FGF23 measurement [21].

Another pitfall concerns the stability of FGF23. Imell and Bacchetta, along with their collaborators, showed consistent results that FGF23 concentrations were undetectable in patients with TIO [22, 23]. There is no reliable explanation for this finding; however, it is suspected that it is a consequence of immunoreactive FGF23 degradation. There is also concern about the direct postvenepuncture instability of FGF23. Dirks et al. demonstrated that the contemporary iFGF23 assays by Immutopics, Kainos, Millipore, and DiaSorin do not require blood withdrawal in protease inhibitor-coated collecting tubes, suggesting that no immediate protein proteolysis occurs after normal blood withdrawal [24]. Furthermore, they proved that the FGF23 concentrations decrease when the centrifugation is delayed up to 8 hours (12% with the Immutopics assay, 7% with the Millipore assay, and 5% with the Kainos assay; all *p* < 0.05). The latter finding confirmed the results of a previous study showing a 23% reduction of FGF23 measured after an 8-hour delay of centrifugation using the second generation of Immutopics [16]. Thus, immediate centrifugation is highly advised.

Several studies have assessed the postcentrifugal stability of intact FGF23 [24–26]. No significant decline was observed in intact FGF23 concentrations, even if the measurements of intact FGF23 in directly centrifugated samples were delayed (8 hours or more). Furthermore, if the processed samples were stored at -80°C, no signs of FGF23 loss were found. In addition, iFGF23 concentrations were also influenced by repeated freeze-thaw cycles (unpublished data). Therefore, samples that have been subjected to repeated freeze-thaw cycles should not be used for further analysis.

### 3.2. Biological Variability

In healthy subjects, a considerable diurnal variation was found in plasma iFGF23 with peak values in the morning as opposed to cFGF23 which increased only modestly throughout the day [27]. Concentrations of cFGF23 appeared to show only a modest, nonsignificant increase during the day [28]. This difference probably reflects inequality in the clearance of the intact protein or its C-terminal fragment [29]. This information has a significant effect on the time of sample collection.

Uncertainty remains concerning the effects of fasting on plasma FGF23 levels. Morning levels in fasting vs. nonfasting samples did not differ remarkably. These results were confirmed by Isakova et al. who found no postprandial changes in plasma cFGF23 concentrations in subjects with early CKD and healthy populations [30]. However, some studies indicate that iFGF23 concentrations increase after phosphate intake with a delay of at least 12 hours [27]. Thus, it is preferable to use fasting or early morning samples to measure iFGF23 levels.

### 3.3. Accuracy and Reproducibility of Methods for FGF23 Measurement

Some publications point to a large variability of FGF23 in diseased persons as opposed to those in full health, as is the case with PTH in haemodialysis patients and for numerous markers in Paget's disease [31–33]. In addition, a significant intraindividual (week-to-week) variation (CVI) of cFGF23 concentration has been reported in patients undergoing haemodialysis [34, 35]. In contrast, according to other dialysis cohorts, CVI of cFGF23 was lower than that of PTH [36, 37]. Smith and colleagues revealed that measurements of plasma FGF23 performed with the cFGF23 assay showed better analytical precision and lower CVI than the iFGF23 assay.

iFGF-23 and cFGF-23 assays differ considerably, and therefore, the results obtained with these methods should not be compared with each other. This was confirmed by Smith et al. that in healthy adults, the reference limits for plasma iFGF-23 concentration are 11.7-48.6 pg/ml and 21.6-91.0 RU/ml for plasma cFGF-23 [27]. Furthermore, they showed poor agreement between plasma iFGF23 and cFGF23 concentrations. Analysis by Passing-Bablok regression found no correlation between iFGF23 and cFGF23 assays as well. Generally, there was no indication to establish a link between these methods.

Research confirms that iFGF23 and cFGF23 assays vary from each other in the intra- and interassay coefficient of variation which are, respectively, <2.4% and <4.7% for the second generation Immutopics, <9.7% and <14% for the Kainos assay [38], <2.9% and <6.3% for the DiaSorin assay [26], and <10% and <8% for the Millipore assay (data from the manufacturer). The Millipore assay is said to have a wider functional analysis range resulting in poor sensitivity at low concentrations [16, 17]. These results indicate that the second generation Immutopics assay is the most reliable and appropriate for FGF23 measurement.

FGF23 concentrations in haemodialysis patients are very high and frequently exceed 100,000 RU/ml. The limited functional analytical range of the available assays is a problem for conditions of FGF23 excess. Therefore, large dilutions may be required in order to bring the concentration within the working range.

### 3.4. Comparison of iFGF23 Assays

Data comparing the analytical agreement between iFGF23 and cFGF23 are available, but information on the correlation of different iFGF23 assays is limited. It was found that current commercial iFGF23 assays show poor analytical agreement (Kainos, Immutopics, Millipore), probably due to differences in calibration [16]. Kainos kits used for measuring plasma iFGF23 concentrations gave higher results than Immutopics or Millipore kits. This disparity increased at higher iFGF23 concentrations. Furthermore, Kainos and Millipore iFGF23 assays showed closer numerical agreement, but not at lower concentrations. Correlation analysis of each iFGF23 and cFGF23 assay indicated no significant association in healthy subjects, in contrast to that observed in haemodialysis patients. In general, the study highlighted that the Kainos assay of the iFGF23 kits evaluated had the most descriptive analytical performance characteristics and Immutopics cFGF23 was the least susceptible to sample instability. Furthermore, results from different assays may not be interchangeable. Unfortunately, the present state of the art excludes a broad clinical benefit of the abovementioned assays.

### 3.5. Intact or C-Terminal Assay: Which One to Choose?

The measurement of cFGF23 has certain advantages over that of iFGF23 in terms of little diurnal variation, more desirable variance characteristics with higher interindividual than intraindividual variation. According to meta-analysis of Xiao et al., C-terminal but not intact FGF23 was significantly associated with all-cause mortality risk [39]. Moreover, cFGF23 is considered a better predictor of CKD progression and atherosclerosis associated with cardiovascular diseases (CVD) and heart failure (HF) [40, 41]. Thus, the cFGF23 assay may be a better choice for clinical use, especially regarding individual risk assessment. Nevertheless, usage of iFGF23 may more precisely represent the biological effect of FGF23. It was reported that FGF23 C-terminal peptides may counteract FGF23's phosphaturic action [29].

### 3.6. Confounding Factors and Diseases

The question of measurement would not be complete without an in-depth analysis of numerous confounding factors, among others: (1) obesity, (2) inflammation, (3) iron deficiency, (4) malnutrition, (5) hyperaldosteronism, and (6) hepatic diseases.

Hu et al. demonstrated that serum FGF23 levels were significantly elevated in obese men and postmenopausal women, especially in those with abdominal obesity [42]. Interestingly, in premenopausal women, no association was found between serum FGF23 levels and either abdominal obesity or overweight/obesity. Mirza et al. reported a positive correlation between serum FGF23 levels and higher body mass index, larger waist circumference, increased triglycerides, lower high-density lipoprotein cholesterol, and elevated total or trunk fat mass [43]. In addition, they confirmed that there is a stronger association between serum FGF23 levels and fat mass in men than in women. Overall, they found evidence that the association between FGF23 and CV risk can be partially mediated by obesity and dyslipidemia, both well-established CVD risk factors.

Inflammation and iron deficiency are common disorders in CKD. Associations among different inflammatory diseases (e.g., acute inflammation/sepsis, rheumatoid arthritis, and childhood inflammatory bowel disease) and FGF23 have already been reported [44–46]. Francis and David revealed that known regulators of FGF23, including calcium, phosphate, and vitamin D, can induce changes that contribute to the increase of FGF23 during inflammation [47]. Data indicate that inflammation increases FGF23 through an iron-related mechanism and a functional iron deficiency can independently stimulate FGF23 production. Schouten et al. reported that the intravenous infusion of iron polymaltose in patients with iron deficiency triggered a temporary increase of FGF23, which almost normalized 3 weeks after infusion [48].

Fukasawa et al. showed that plasma FGF23 concentrations correlated positively with muscle mass indices in haemodialysis patients [49]. They claimed that FGF23 and nutritional status are highly associated with each other. Minoo et al. conducted a study among the patients with end-stage renal disease receiving haemodialysis maintenance therapy [50]. They concluded that FGF23 had a linear association with vitamin D and that its increase positively correlated with the increase of vitamin D. However, the association between mineral factors, PTH and FGF23, was not confirmed. These results suggest that probably not all factors that induce skeletal expression of phosphate are yet fully known.

The effect of hyperaldosteronism on the release of FGF23 has also been a focus of studies. Zhang et al. conducted a study in mice divided into three groups: the first group receiving sham treatment, the second treated with a single subcutaneous injection of deoxycorticosterone, and the third with spironolactone [51]. The observation was that mineralocorticoids can influence the regulation of FGF23 transcription and release in vivo. Previous research has already shown that the release of FGF23 is caused by an increase in the cytosolic Ca2+ concentration, which in turn is triggered by store-operated Ca2+ entry (SOCE) [52]. Both aldosterone and spironolactone reduced the SOCE modifying transcription of FGF23. In summary, the expression of FGF23 is upregulated by aldosterone.

A French study revealed that patients with autosomal dominant polycystic kidney disease (ADPKD) with preserved renal function had higher cFGF23 levels than other patients with CKD [53]. The likely explanation is that highly polycystic liver produces FGF23 and increases FGF23 levels in patients with ADPKD.

It is reported that a growing number of diseases induce an increase in FGF23. The question therefore arises whether FGF23 exerts some biological function.

## 4. FGF23-Related Diseases

The physiological effect of FGF23 in phosphate and vitamin D metabolism has been confirmed by studies on a few hereditary diseases that lead either to an excess or an insufficiency of bioactive FGF23 [54, 55]. The FGF23 bone-kidney axis is a newly discovered regulatory system that is integrated with the PTH vitamin D axis and plays an equally important role in the health and development of some diseases.

### 4.1. Hypophosphatemic Disorders

Rickets and osteomalacia are associated with a disturbed mineralization of the bone matrix, which leads to a softening of the bones. Rickets develops in children and typically causes growth retardation and bent legs [56–58]. Adults may be affected by a similar condition called osteomalacia, which causes muscle weakness and bone pain. Despite the fact that rickets and osteomalacia have a different etiology, they are usually caused by chronic hypophosphatemia.

There are several medical conditions that cause hypophosphatemic rickets/osteomalacia and have a common clinical manifestation. These are three genetic diseases: ADHR, autosomal recessive hypophosphatemic rickets/osteomalacia (ARHR), XLH, a paraneoplastic syndrome-TIO, and hypophosphatemic rickets/osteomalacia associated with McCune Albright Syndrome (MAS)/fibrous dysplasia (FD). Patients suffering from these diseases have a phosphate reabsorption defect in the proximal tubules. Consequently, an increased 1,25(OH)_2_D production should have been observed. However, in these FGF23-related hypophosphatemic rickets/osteomalacia, the concentration of 1,25(OH)_2_D remains normal or low. Therefore, it is assumed that these hypophosphatemic diseases are based on both impaired phosphate reabsorption and abnormal vitamin D metabolism. The mechanism of FGF23 hyperactivity is differentiated (see Table 1).

FGF23: fibroblast growth factor 23; PHEX: phosphate-regulating gene with homologies to endopeptidases on the X chromosome; DMP1: dentin matrix protein 1.

### 4.2. Hyperphosphatemic Disorders

Tumor calcification (TC) is a rare clinically pathological entity characterized by ectopic calcification, mainly around large joints. This condition is most commonly observed in patients with end-stage renal disease [56, 59]. However, there are several types of TC in patients with preserved renal function. Hyperphosphatemic familial neoplastic calcinosis (HFTC) is an autosomal recessive disease with an unreasonable increase in renal tubular phosphate reabsorption and elevated or normal levels of 1,25 (OH)_2_D. Numerous studies suggest that genetic defects lead to a reduction in intact FGF23 levels or a deterioration in the function of FGF23. This hyperphosphatemic disease has been shown to be caused by mutations in three genes UDP-N-acetyl-alpha-D-galactosamine: N-acetylgalactosaminyltransferase 3 (GALNT3) polypeptide; FGF23 and Klotho [60–63].

Yamashita et al. showed that one generation of FGF23-null mice showed severe hyperphosphatemia with increased renal phosphate reabsorption and a high concentration of 1,25(OH)_2_D [64]. They also showed that the clinical and biochemical phenotype of mutant mice and TC patients is similar.

Elevated levels of FGF23 are also found in patients with hyperparathyroidism. It is believed that elevated levels of FGF23 can compensate for the hyperphosphatemia or phosphate overload in these diseases. It is not known whether any other factor could contribute to the increase in FGF23 levels.

## 5. FGF23 and Chronic Kidney Disease

In the course of CKD, one of the first metabolic disorders is phosphate retention [65]. CKD is also believed to be one of the most common diseases with high levels of FGF-23. The increase in FGF23 plasma levels occurs before the increase in PTH and phosphate (PO_4_^3-^) and the decrease in 1,25(OH)_2_D, calcium (Ca) concentrations [66]. This may be a compensatory mechanism responsible for maintaining physiological phosphate levels until the final stage of kidney disease when the phosphate balance is compromised. Anomalies in Ca and PO_4_^3-^ metabolism are frequently observed in hemodialysed patients. Multiple publications suggest that these changes are an early complication in patients with CKD and that they are considered a serious risk factor for both renal dysfunction and CVD [67, 68].

FGF23 is one of the Ca and PO_4_^3-^ metabolism regulators that reduces phosphate reabsorption in the renal tubules [66–71]. It is recognized that this factor is closely related to progressive renal dysfunction, CVD complications, and secondary hyperparathyroidism (HPT). FGF23 may also play a protective role against toxic phosphate concentration. However, it is still to be determined whether FGF23 is a protective protein or whether it could be one of the uremic toxins.

Alterations in mineral metabolism in CKD are described as chronic kidney disease-Mineral Bone Disorder (CKD-MBD). This syndrome is responsible for an increased risk of CVD including mortality in patients with CKD [1]. Recent studies have shown that FGF23 may be considered a CVD risk factor in CKD-MBD [72, 73].

Vitamin D deficiency may trigger renin-angiotensin-aldosterone system (RAAS) overactivation as a mechanism underlying the exacerbation of CKD and CVD [74]. The activation of the RAAS reduces the renal expression of klotho, which is essential for proper FGF23 signaling. Consequently, high FGF23 levels contribute to a further decrease in vitamin D. Finally, such a feedback loop leads to activation of the RAAS, high FGF23 levels, vitamin D, and renal klotho deficiency.

## 6. FGF23 in Cardiology

Whereas FGF23 in low levels most likely does not affect the cardiovascular system, higher levels may be associated with endothelial dysfunction and RAAS activation [75, 76]. In one mouse study, FGF23 was found to directly inhibit endothelium-dependent vasorelaxation by increasing superoxide levels and reducing nitric oxide bioavailability [77]. Another series pointed to an association between higher circulating FGF23 and vascular dysfunction in patients with an estimated glomerular filtration rate (eGFR) ≥90 ml/min/1.73 m^2^, whereas an association with increased arterial stiffness was found exclusively in those with eGFR <60 ml/min/1.73 m^2^ [78].

**Figure 2:**
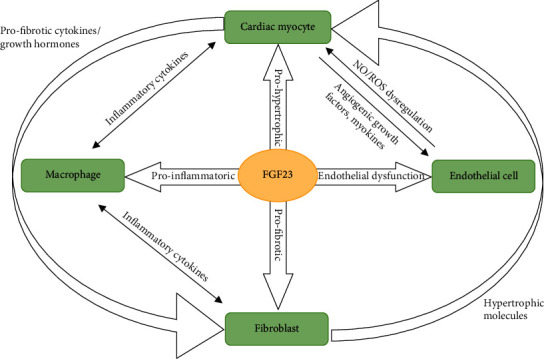
The relationship between various cells type. FGF23 is produced by cardiomyocytes, fibroblasts, endothelial cells, and macrophages. FGF23 directly induces prohypertrophic, profibrotic, and proinflammatory signaling via paracrine mechanisms. FGF23: fibroblast growth factor 23; NO: nitric oxide; ROS: reactive oxygen species.

Cardiomyocytes may promote cardiac remodeling via autocrine and/or paracrine mechanisms (see Figure 2). It is shown that patients with CKD have excessive cardiomyocyte production of FGF23 leading to upregulation of FGFR4 and activation of the calcineurin-NFAT pathway. This, together with a reduced renal expression of the FGF23 coreceptor Klotho induce cardiac remodeling and left ventricular hypertrophy (LVH) in patients on dialysis but not in those with a functioning kidney transplant [79]. Furthermore, myocardial fibrosis may be induced in mice after myocardial infarction (MI) or ischemia/reperfusion by overexpression of FGF23 associated with upregulation of *β*-catenin, TGF-*β*, and procollagen I and III [80].

Wohlfahrt et al. suggested that FGF23 is a strong predictor of adverse events (including death of all causes, urgent heart transplantation, or implantation of a ventricular assist device) in patients with chronic HF and nonobvious CKD [81]. Interestingly, they identified a group of HF patients with elevated FGF23 levels who could benefit more from angiotensin-converting enzyme inhibitors therapy.

In the literature, one may find rather poor and contradictory data concerning the relationship between FGF23 and coronary artery disease (CAD). Large epidemiological studies that investigated FGF23 levels in relation to CVD risk and mortality showed a strong association, especially with HF and stroke [82, 83], but hardly any with CAD including MI. However, most of these studies were conducted in patients with preexisting CAD and in elderly people. Therefore, the evidence remains unclear for patients without CAD, which calls for further investigation.

Udell et al. measured FGF23 in patients with stable CAD and followed them up over a median of 5.1 years [84]. This is one of the first studies to indicate that increased FGF23 levels are associated with adverse CV risk (CV death, incident HF) independent of renal function. However, the researchers found no significant trends associating FGF23 levels in the highest quartile and the risk of MI and stroke. While the major strengths of this study are its robust sample size and the number of registered clinical events, the limitations are already established CAD and lack of data on PTH, Ca, PO_4_^3-^, or vitamin D levels. The Heart and Soul Study included outpatients with CAD and showed that elevated FGF23 levels correlated significantly with mortality and CVD events independent of other CVD risk factors, renal function, and C-reactive protein levels [85]. Despite impressive results, bear in mind that participants included in the study had prevalent CAD, and most were older men.

Interestingly, Bergmark et al. evaluated the relevance of elevated FGF23 levels after acute coronary syndrome (ACS) [86] and showed that FGF23 provides prognostic information that is superior to already establish predictors and biomarkers, such as natriuretic peptide, eGFR, or highly sensitive troponin I. Furthermore, they demonstrated that higher FGF23 levels are associated with undesirable CV outcomes such as CV death and HF hospitalization. The study appears to be well-designed with a large sample size, number of events, and adjudicated outcomes. Even if left ventricular ejection fraction (LVEF) was only measured in 84% of patients, the results were consistent after the inclusion of LVEF in the adjustment model. Recently, Austrian researchers confirmed that elevated FGF23 levels are clearly and independently associated with the development of left ventricular remodeling in patients with ST elevation myocardial infarction (STEMI) treated with primary percutaneous coronary intervention [54]. Furthermore, they emphasized that the use of a multimarker strategy, including FGF23, may improve risk assessment in patients with STEMI. Pöss et al. measured FGF23 in patients with infarction-related cardiogenic shock (CS) with the thrombolysis in myocardial infarction (TIMI) 3 flow after acute coronary intervention [87]. They showed that patients with the highest FGF23 concentrations had a worse outcome and that FGF23 can be used as an independent predictor of short-term mortality.

Another large study showed that FGF23 concentrations correlate significantly with CAD incidents, HF, and CV mortality [88]. Furthermore, an additional adjustment for markers of renal function weakened the associations only slightly. While the major strengths of this study are a biracial population-based sample, a large number of events, and corresponding power for subgroup analyses, a serious limitation is that FGF23 was measured in singlicate that may result in regression dilution bias. Spanish researchers conducted the studies which included patients with non-ST elevation myocardial infarction (NSTEMI) or STEMI [89, 90]. This study was the first to demonstrate that the simultaneous assessment of calcidiol and FGF23 levels was a strong predictor of adverse events in patients with CAD. Furthermore, the study showed that low eGFR does not influence the results. The same clinicians performed further analysis to assess whether seasonal variations in sun exposure could affect calcidiol and FGF23 levels. They found that an adverse outcome can be predicted in patients with stable CAD, low calcidiol, and high FGF23 levels.

Numerous studies suggest that FGF23 is a strong predictor for HF decompensation. High levels of FGF23 are to be associated with mortality and LVH in dialysis patients and with the development of atherosclerosis in elderly people [91–93]. Gruson et al. showed that the measurement of FGF23 provides an added value to natriuretic peptide for risk assessment (multiple biomarker strategy) in HF patients with reduced LVEF [94]. Furthermore, they showed that FGF23 concentrations are associated with an increased risk of long-term CV death.

Several medical studies suggest that FGF23 can also be regarded as a marker for vascular calcification. Srivaths et al. reported that higher FGF23 levels correlate significantly with both coronary calcifications and final Agatston score [95]. There is a theory that it is due to reduced Klotho levels that are associated with LVH, myocardial fibrosis, and increased mortality [96, 97]. According to Bergmark et al., a combination of low Klotho and elevated FGF23 may serve as a contributing marker of increased CV risk [98].

A few publications indicate also that there is a significant association between FGF23 concentrations and the most common cardiac arrhythmia, atrial fibrillation (AF). Patients with AF had considerably higher levels of FGF23, independently of classic risk factors or renal failure [99]. Chua et al. performed a study and quantified 40 common CV biomarkers [100]. The research revealed that two biomarkers, including FGF23 and brain natriuretic peptide (BNP) as well as assessment of three clinical risk factors: age, sex, and body mass index, may unambiguously identify patients with AF.

Despite numerous investigations, the exact role of FGF23 in the pathogenesis and course of CVD remains unclear. An important question arises here: Do the elevated FGF23 levels among people without previous history of CKD indicate increased CV risk? Further large-scale trials are required to examine this issue. Confirmation of FGF23's predictive value might contribute to the early identification of patients with high CV risk and further diagnostic accuracy improvement. Perhaps, FGF23 may become an easy and fast predictive tool in the future.

## 7. Clinical Impact of FGF23

Measurement of FGF23 can help in diagnosis and further management of abnormalities of phosphate and bone metabolism among patients with either preserved renal function or overt kidney failure. Nevertheless, CKD appears to be the main cause of the secondary increase in FGF23 levels. FGF23 plays a pivotal role in maintaining phosphorus balance in CKD (state of phosphorus excess) with a subsequent decline of 1,25(OH)2D supporting the view that elevated FGF23 may underly the genesis of secondary HPT. In recent years, FGF23 has gained wide attention in CKD and emerged as a promising biomarker for predicting adverse outcomes in different stages of CKD patients.

Current data indicate also that high FGF-23 levels are associated with adverse clinical events including CVD and mortality. Such results are not surprising since FGF-23 is linked to the development of LVH, CAD and MI, stroke, and impairment of immune response. Therefore, FGF-23 should be considered a uremic toxin [101].

## 8. Therapeutic Potential of FGF23

A large-scale trial was to determine the benefits and risks of calcimimetic cinacalcet in terms of reducing FGF23 levels in patients with terminal renal failure [102]. The study showed that cinacalcet significantly reduced serum FGF23 and CV mortality risk and was associated with fewer CV events such as HF and sudden death.

Yamazaki et al. used two monoclonal antibodies, namely, FN1 and FC1, which can neutralize the effect of FGF23 [103]. They reported that FC1 and FN1 block the association of FGF23 with its FGFR and *α*-klotho, respectively. They observed also that a single injection of FN1 or FC1 caused only a significant elevation in serum phosphate and vitamin D. Finally, they confirmed that the neutralizing antibodies can be used to develop new therapeutic strategies for FGF23-related diseases.

Stark warning came with an American publication evaluating treatment with FGF23-neutralizing antibodies in secondary HPT and subsequent analysis of its effect on CKD progression in rats [104]. Shalhoub et al. showed that chronic neutralization of FGF23 was associated with several beneficial effects, such as increased vitamin D levels, decreased HPT, and other improved bone parameters. Unfortunately, blocking the phosphaturic activity of FGF23 led to increased mortality and vascular calcification. Furthermore, the use of antibodies did not prevent the development of LVH in animals.

## 9. State of Uncertainty and Recommendation for Future Research

FGF23 is a promising biomarker linking CKD with CV morbidity and mortality. The FGF23–klotho axis and pathophysiological cardiac effects are complex and far from being fully understood. The precise roles of klotho, FGFRs, and local and systemic FGF23 also need to be determined. Furthermore, regulatory mechanisms and determinants of FGF23 production or the PTH mechanism of action on phosphate excretory function of FGF23 still remain enigmatic.

There are many more questions to be answered regarding FGF23 effects upon cardiac structure and function: whether FGF23 secretion causes heart diseases or vice versa, whether FGF23 plays a special role in acute MI cases, and whether cardiomyocytes produce FGF23 in health and disease, for example.

Another concern is whether any intervention can modify FGF23 levels and whether this may be associated with improved outcomes. A few scientists consider FGF23 a therapeutic target. However, before a therapeutic strategy can be implemented, further large-scale studies are necessary to clarify fundamental unresolved questions. If studies on therapies aiming at a reduction of the bioactivity of FGF23 are successful, a routine measurement of FGF23 may appear useful. However, there is an urgent need for standardization of commercial assays for FGF23 and the introduction of an international standard. The collection of further data on the cleavage of FGF23 and the biological effects of the fragments would also be of crucial importance.

## 10. Conclusions

FGF23 plays a crucial role in calcium-phosphate metabolism regulation and interorgan signaling. Currently, much evidence points to the fact that FGF23 is not just an innocent bystander, but rather a pathophysiological factor in various diseases. Many clinical trials already show not only that elevated FGF23 levels are independently associated with faster progression of CKD, LVH, but also an increased CV mortality. Nevertheless, the influence of FGF23 on CV disorders has not been fully elucidated. To establish FGF23 as a target for improving CV outcomes, further studies on the underlying pathophysiological mechanisms are needed. The present review gives also attention to the reliability of FGF23 measurement and potential use of FGF23 as a target for therapy. Perhaps, FGF23 may appear a key factor in various fields of medicine, especially nephrology and cardiology.

## Figures and Tables

**Table 1:** Hypophosphatemic diseases caused by FGF23 overproduction and hyperactivity.

Disease	Cause
Autosomal dominant hypophosphatemic rickets/osteomalacia	Dysregulated expression of FGF23 by mutations in the FGF23 gene
Autosomal recessive hypophosphatemic rickets/osteomalacia	Overexpression of FGF23 in bone by mutation in the DMP1 gene
X-linked hypophosphatemic rickets/osteomalacia	Overexpression of FGF23 in bone by mutation in the PEHX gene
McCune-Albright syndrome/fibrous dysplasia	Overexpression of FGF23 in bone
Tumor-induced rickets/osteomalacia	Overexpression of FGF23 in responsible tumors

## Data Availability

The data supporting this review are from previously reported studies and datasets, which have been cited at relevant places within the text as references [1–104].

## References

[1] Martin A., David V., Quarles L. D. (2012). Regulation and function of the FGF23/klotho endocrine pathways. *Physiological Reviews*.

[2] Yamashita T., Yoshioka M., Itoh N. (2000). Identification of a novel fibroblast growth factor, FGF-23, preferentially expressed in the ventrolateral thalamic nucleus of the brain. *Biochemical and Biophysical Research Communications*.

[3] ADHR Consortium (2000). Autosomal dominat hypophosphatemic rickets is associated with mutations in FGF23. *Nature Genetics*.

[4] Benet-Pages A., Orlik P., Strom T. M., Lorenz-Depiereux B. (2005). An FGF23 missense mutation causes familial tumoral calcinosis with hyperphosphatemia. *Human Genetics*.

[5] Gutiérrez O. M., Mannstadt M., Isakova T. (2008). Fibroblast growth factor 23 and mortality among patients undergoing hemodialysis. *The New England Journal of Medicine*.

[6] Jean G., Terrat J. C., Vanel T. (2009). High levels of serum fibroblast growth factor (FGF)-23 are associated with increased mortality in long haemodialysis patients. *Nephrology, Dialysis, Transplantation*.

[7] Souma N., Isakova T., Lipiszko D. (2016). Fibroblast growth factor 23 and cause-specific mortality in the general population: the northern Manhattan study. *Journal of Clinical Endocrinology and Metabolism*.

[8] Lang F., Leibrock C., Pandyra A., Stournaras C., Wagner C. A., Föller M. (2018). Phosphate homeostasis, inflammation and the regulation of FGF-23. *Kidney & Blood Pressure Research*.

[9] Kuro-o M., Matsumura Y., Aizawa H. (1997). Mutation of the mouse klotho gene leads to a syndrome resembling ageing. *Nature*.

[10] Bouma-de Krijger A., Vervloet M. G. (2020). Fibroblast growth factor 23: are we ready to use it in clinical practice?. *Journal of Nephrology*.

[11] Gattineni J., Bates C., Twombley K. (2009). FGF23 decreases renal NaPi-2a and NaPi-2c expression and induces hypophosphatemia in vivo predominantly via FGF receptor 1. *American Journal of Physiology. Renal Physiology*.

[12] Nissenson R. A., Juppner H.

[13] Watts N. B., Rosen C. J. (2013). Estrogens, Estrogen agonists/antagonists, and calcitonin. *Primer on the Metabolic Bone Diseases and Disorders of Mineral Metabolism*.

[14] Ben-Dov I. Z., Galitzer H., Lavi-Moshayoff V. (2007). The parathyroid is a target organ for FGF23 in rats. *The Journal of Clinical Investigation*.

[15] Liu S., Quarles L. D. (2007). How fibroblast growth factor 23 works. *Journal of the American Society of Nephrology*.

[16] Smith E. R., McMahon L. P., Holt S. G. (2013). Method-specific differences in plasma fibroblast growth factor 23 measurement using four commercial ELISAs. *Clinical Chemistry and Laboratory Medicine*.

[17] Sinha M. D., Turner C., Goldsmith D. J. (2013). FGF23 concentrations measured using “Intact” assays similar but not interchangeable. *International Urology and Nephrology*.

[18] Shimizu Y., Fukumoto S., Fujita T. (2012). Evaluation of a new automated chemiluminescence immunoassay for FGF23. *Journal of Bone and Mineral Metabolism*.

[19] Fassbender W. J., Brandenburg V., Schmitz S. (2009). Evaluation of human fibroblast growth factor 23 (FGF-23) C-terminal and intact enzyme-linked immunosorbent-assays in end-stage renal disease patients. *Clinical Laboratory*.

[20] Smith E. R., McMahon L. P., Holt S. G. (2014). Fibroblast growth factor 23. *Annals of Clinical Biochemistry*.

[21] Damasiewicz M. J., Lu Z. X., Kerr P. G., Polkinghorne K. R. (2018). The stability and variability of serum and plasma fibroblast growth factor-23 levels in a haemodialysis cohort. *BMC Nephrology*.

[22] Imel E. A., Peacock M., Pitukcheewanont P. (2006). Sensitivity of fibroblast growth factor 23 measurements in tumor-induced osteomalacia. *The Journal of Clinical Endocrinology and Metabolism*.

[23] Bacchetta J., Dubourg L., Harambat J. (2010). The influence of glomerular filtration rate and age on fibroblast growth factor 23 serum levels in pediatric chronic kidney disease. *The Journal of Clinical Endocrinology and Metabolism*.

[24] Dirks N. F., Smith E. R., van Schoor N. M. (2019). Pre-analytical stability of FGF23 with the contemporary immunoassays. *Clinica Chimica Acta*.

[25] El-Maouche D., Dumitrescu C. E., Andreopoulou P. (2016). Stability and degradation of fibroblast growth factor 23 (FGF23): the effect of time and temperature and assay type. *Osteoporosis International*.

[26] Souberbielle J. C., Prie D., Piketty M. L. (2017). Evaluation of a new fully automated assay for plasma intact FGF23. *Calcified Tissue International*.

[27] Smith E. R., Cai M. M., McMahon L. P., Holt S. G. (2012). Biological variability of plasma intact and C-terminal FGF23 measurements. *The Journal of Clinical Endocrinology and Metabolism*.

[28] Vervloet M. G., van Ittersum F. J., Buttler R. M., Heijboer A. C., Blankenstein M. A., ter Wee P. M. (2011). Effects of dietary phosphate and calcium intake on fibroblast growth factor-23. *Clinical Journal of the American Society of Nephrology*.

[29] Goetz R., Nakada Y., Hu M. C. (2010). Isolated C-terminal tail of FGF23 alleviates hypophosphatemia by inhibiting FGF23–FGFR–klotho complex formation. *Proceedings of the National Academy of Sciences of the United States of America*.

[30] Isakova T., Gutierrez O., Shah A. (2008). Postprandial mineral metabolism and secondary hyperparathyroidism in early CKD. *Journal of the American Society of Nephrology*.

[31] Ricós C., Iglesias N., García-Lario J. V. (2007). Within subject biological variation in disease: collated data and clinical consequences. *Annals of Clinical Biochemistry*.

[32] Fischer D. C., Mischek A., Wolf S. (2012). Paediatric reference values for the C-terminal fragment of fibroblast growth factor-23, sclerostin, bone-specific alkaline phosphatase and isoform 5b of tartrate-resistant acid phosphatase. *Annals of Clinical Biochemistry*.

[33] Alvarez L., RicÓs C., Peris P. (2000). Components of biological variation of biochemical markers of bone turnover in Paget's bone disease. *Bone*.

[34] Cavalier E., Delanaye P., Moranne O. (2013). Variability of new bone mineral metabolism markers in patients treated with maintenance hemodialysis: implications for clinical decision making. *American Journal of Kidney Diseases*.

[35] Seiler S., Lucisano G., Ege P. (2013). Single FGF-23 measurement and time-averaged plasma phosphate levels in hemodialysis patients. *Clinical Journal of the American Society of Nephrology*.

[36] Isakova T., Xie H., Barchi-Chung A. (2011). Fibroblast growth factor 23 in patients undergoing peritoneal dialysis. *Clinical Journal of the American Society of Nephrology*.

[37] Jia T., Qureshi A. R., Brandenburg V. (2013). Determinants of fibroblast growth factor-23 and parathyroid hormone variability in dialysis patients. *American Journal of Nephrology*.

[38] Heijboer A. C., Levitus M., Vervloet M. G. (2009). Determination of fibroblast growth factor 23. *Annals of Clinical Biochemistry*.

[39] Xiao Y., Luo X., Huang W., Zhang J., Peng C. (2014). Fibroblast growth factor 23 and risk of all-cause mortality and cardiovascular events: a meta-analysis of prospective cohort studies. *International Journal of Cardiology*.

[40] Fliser D., Kollerits B., Neyer U. (2007). Fibroblast growth factor 23 (FGF23) predicts progression of chronic kidney disease: the mild to moderate kidney disease (MMKD) study. *Journal of the American Society of Nephrology*.

[41] Emrich I. E., Brandenburg V., Sellier A. B. (2019). Strength of fibroblast growth factor 23 as a cardiovascular risk predictor in chronic kidney disease weaken by ProBNP adjustment. *American Journal of Nephrology*.

[42] Hu X., Ma X., Luo Y. (2018). Associations of serum fibroblast growth factor 23 levels with obesity and visceral fat accumulation. *Clinical Nutrition*.

[43] Mirza M. A., Alsiö J., Hammarstedt A. (2011). Circulating fibroblast growth factor-23 is associated with fat mass and dyslipidemia in two independent cohorts of elderly individuals. *Arteriosclerosis, Thrombosis, and Vascular Biology*.

[44] Dounousi E., Torino C., Pizzini P. (2016). Intact FGF23 and *α*-Klotho during acute inflammation/sepsis in CKD patients. *European Journal of Clinical Investigation*.

[45] Sato H., James Kazama J., Murasawa A. (2016). Serum fibroblast growth factor 23 (FGF23) in patients with rheumatoid arthritis. *Internal Medicine*.

[46] el-Hodhod M. A. A., Hamdy A. M., Abbas A. A., Moftah S. G., Ramadan A. A. (2012). Fibroblast growth factor 23 contributes to diminished bone mineral density in childhood inflammatory bowel disease. *BMC Gastroenterology*.

[47] Francis C., David V. (2016). Inflammation regulates fibroblast growth factor 23 production. *Current Opinion in Nephrology and Hypertension*.

[48] Schouten B. J., Hunt P. J., Livesey J. H., Frampton C. M., Soule S. G. (2009). FGF23 elevation and hypophosphatemia after intravenous iron polymaltose: a prospective study. *The Journal of Clinical Endocrinology and Metabolism*.

[49] Fukasawa H., Ishigaki S., Kinoshita-Katahashi N. (2014). Plasma levels of fibroblast growth factor-23 are associated with muscle mass in haemodialysis patients. *Nephrology (Carlton)*.

[50] Minoo F., Ramezanzade E., Mojarad M., Alamdari A., Najafi M. T. (2018). The association of fibroblast growth factor-23 with mineral factors (Ca, P, and Mg), parathyroid hormone, and 25-hydroxyvitamin D in hemodialysis patients: a multicenter study. *Nephro-Urology Monthly*.

[51] Zhang B., Umbach A. T., Chen H. (2016). Up-regulation of FGF23 release by aldosterone. *Biochemical and Biophysical Research Communications*.

[52] Zhang B., Yan J., Umbach A. T. (2016). NFkB-sensitive Orai1 expression in the regulation of FGF23 release. *Journal of Molecular Medicine*.

[53] Bienaimé F., Ambolet A., Aussilhou B. (2018). Hepatic production of fibroblast growth factor 23 in autosomal dominant polycystic kidney disease. *The Journal of Clinical Endocrinology & Metabolism*.

[54] Quarles L. D. (2003). FGF23, PHEX, and MEPE regulation of phosphate homeostasis and skeletal mineralization. *American Journal of Physiology-Endocrinology and Metabolism*.

[55] Strom T. M., Jüppner H. (2008). PHEX, FGF23, DMP1 and beyond. *Current Opinion in Nephrology and Hypertension*.

[56] Saito T., Fukumoto S. (2009). Fibroblast growth factor 23 (FGF23) and disorders of phosphate metabolism. *International Journal of Pediatric Endocrinology*.

[57] Chehade H., Girardin E., Rosato L., Cachat F., Cotting J., Perez M. H. (2011). Acute life-threatening presentation of vitamin D deficiency rickets. *The Journal of Clinical Endocrinology and Metabolism*.

[58] Fukumoto S. (2008). Physiological regulation and disorders of phosphate metabolism - pivotal role of fibroblast growth factor 23. *Internal Medicine*.

[59] Lyles K. W., Halsey D. L., Friedman N. E., Lobaugh B. (1988). Correlations of serum concentrations of 1,25-dihydroxyvitamin D, phosphorus, and parathyroid hormone in tumoral calcinosis. *Journal of Clinical Endocrinology and Metabolism*.

[60] Topaz O., Shurman D. L., Bergman R. (2004). Mutations in GALNT3, encoding a protein involved in O-linked glycosylation, cause familial tumoral calcinosis. *Nature Genetics*.

[61] Larsson T., Davis S. I., Garringer H. J. (2005). Fibroblast growth factor-23 mutants causing familial tumoral calcinosis are differentially processed. *Endocrinology*.

[62] Araya K., Fukumoto S., Backenroth R. (2005). A novel mutation in fibroblast growth factor 23 gene as a cause of tumoral calcinosis. *Journal of Clinical Endocrinology and Metabolism*.

[63] Ichikawa S., Imel E. A., Kreiter M. L. (2007). A homozygous missense mutation in human KLOTHO causes severe tumoral calcinosis. *Journal of Clinical Investigation*.

[64] Shimada T., Kakitani M., Yamazaki Y. (2004). Targeted ablation of Fgf23 demonstrates an essential physiological role of FGF23 in phosphate and vitamin D metabolism. *The Journal of Clinical Investigation*.

[65] Slatopolsky E., Caglar S., Pennell J. P. (1971). On the pathogenesis of hyperparathyroidism in chronic experimental renal insufficiency in the dog. *The Journal of Clinical Investigation*.

[66] Isakova T., Wahl P., Vargas G. S. (2011). Fibroblast growth factor 23 is elevated before parathyroid hormone and phosphate in chronic kidney disease. *Kidney International*.

[67] Block G., Port F. K. (2003). Calcium phosphate metabolism and cardiovascular disease in patients with chronic kidney disease. *Seminars in Dialysis*.

[68] Kestenbaum B., Belozeroff V. (2007). Mineral metabolism disturbances in patients with chronic kidney disease. *European Journal of Clinical Investigation*.

[69] Isakova T., Xie H., Yang W. (2011). Fibroblast growth factor 23 and risks of mortality and end-stage renal disease in patients with chronic kidney disease. *JAMA*.

[70] Kendrick J., Cheung A. K., Kaufman J. S. (2011). FGF-23 associates with death, cardiovascular events, and initiation of chronic dialysis. *Journal of the American Society of Nephrology*.

[71] Seiler S., Reichart B., Roth D., Seibert E., Fliser D., Heine G. H. (2010). FGF-23 and future cardiovascular events in patients with chronic kidney disease before initiation of dialysis treatment. *Nephrology, Dialysis, Transplantation*.

[72] Goldfarb M., Schoorlemmer J., Williams A. (2007). Fibroblast growth factor homologous factors control neuronal excitability through modulation of voltage-gated sodium channels. *Neuron*.

[73] Gupta A., Winer K., Econs M. J., Marx S. J., Collins M. T. (2004). FGF-23 is elevated by chronic hyperphosphatemia. *The Journal of Clinical Endocrinology and Metabolism*.

[74] Melamed M. L., Astor B., Michos E. D., Hostetter T. H., Powe N. R., Muntner P. (2009). 25-hydroxyvitamin D levels, race, and the progression of kidney disease. *Journal of the American Society of Nephrology*.

[75] Yilmaz M. I., Sonmez A., Saglam M. (2010). FGF-23 and vascular dysfunction in patients with stage 3 and 4 chronic kidney disease. *Kidney International*.

[76] Böckmann I., Lischka J., Richter B. (2019). FGF23-mediated activation of local RAAS promotes cardiac hypertrophy and fibrosis. *International Journal of Molecular Sciences*.

[77] Silswal N., Touchberry C. D., Daniel D. R. (2014). FGF23 directly impairs endothelium-dependent vasorelaxation by increasing superoxide levels and reducing nitric oxide bioavailability. *American Journal of Physiology. Endocrinology and Metabolism*.

[78] Mirza M. A., Larsson A., Lind L., Larsson T. E. (2009). Circulating fibroblast growth factor-23 is associated with vascular dysfunction in the community. *Atherosclerosis*.

[79] Leifheit-Nestler M., große Siemer R., Flasbart K. (2016). Induction of cardiac FGF23/FGFR4 expression is associated with left ventricular hypertrophy in patients with chronic kidney disease. *Nephrology, Dialysis, Transplantation*.

[80] Hao H., Li X., Li Q. (2016). FGF23 promotes myocardial fibrosis in mice through activation of *β*-catenin. *Oncotarget*.

[81] Wohlfahrt P., Melenovsky V., Kotrc M. (2015). Association of fibroblast growth factor-23 levels and angiotensin-converting enzyme inhibition in chronic systolic heart failure. *JACC: Heart Failure*.

[82] Ix J. H., Katz R., Kestenbaum B. R. (2012). Fibroblast growth factor-23 and death, heart failure, and cardiovascular events in community-living individuals: CHS (Cardiovascular Health Study). *Journal of the American College of Cardiology*.

[83] Udell J., O'Donnell T., Morrow D. (2012). Association of fibroblast growth factor (FGF)-23 levels with risk of cardiovascular events in patients with stable coronary artery disease. *Journal of the American College of Cardiology*.

[84] Udell J. A., Morrow D. A., Jarolim P. (2014). Fibroblast growth factor-23, cardiovascular prognosis, and benefit of angiotensin-converting enzyme inhibition in stable ischemic heart disease. *Journal of the American College of Cardiology*.

[85] Parker B. D., Schurgers L. J., Brandenburg V. M. (2010). The associations of fibroblast growth factor 23 and uncarboxylated matrix Gla protein with mortality in coronary artery disease: the heart and soul study. *Annals of Internal Medicine*.

[86] Bergmark B. A., Udell J. A., Morrow D. A. (2018). Association of fibroblast growth factor 23 with recurrent cardiovascular events in patients after an acute coronary syndrome: a secondary analysis of a randomized clinical trial. *JAMA Cardiology*.

[87] Reindl M., Reinstadler S. J., Feistritzer H. J. (2017). Fibroblast growth factor 23 as novel biomarker for early risk stratification after ST-elevation myocardial infarction. *Heart*.

[88] Pöss J., Mahfoud F., Seiler S. (2013). FGF-23 is associated with increased disease severity and early mortality in cardiogenic shock. *European Heart Journal Acute Cardiovascular Care*.

[89] Lutsey P. L., Alonso A., Selvin E. (2014). Fibroblast growth factor-23 and incident coronary heart disease, heart failure, and cardiovascular mortality: the atherosclerosis risk in communities study. *Journal of the American Heart Association*.

[90] Tuñón J., Cristóbal C., Tarín N. (2014). Coexistence of low vitamin D and high fibroblast growth factor-23 plasma levels predicts an adverse outcome in patients with coronary artery disease. *PLoS One*.

[91] Aceña A., Franco-PelÁez J., Gutierrez-Landaluce C. (2017). Sun exposure influences the prognostic power of components of mineral metabolism in patients with coronary artery disease. *Metabolism and Cardiovascular Diseases*.

[92] Gutiérrez O. M., Januzzi J. L., Isakova T. (2009). Fibroblast growth factor 23 and left ventricular hypertrophy in chronic kidney disease. *Circulation*.

[93] Mirza M. A. I., Hansen T., Johansson L. (2009). Relationship between circulating FGF23 and total body atherosclerosis in the community. *Nephrology, Dialysis, Transplantation*.

[94] Gruson D., Carbone V., Feracin B., Ahn S. A., Rousseau M. F. (2018). Incremental value of intact fibroblast growth factor 23 to natriuretic peptides for long-term risk estimation of heart failure patients. *Clinical Biochemistry*.

[95] Srivaths P. R., Goldstein S. L., Krishnamurthy R., Silverstein D. M. (2014). High serum phosphorus and FGF 23 levels are associated with progression of coronary calcifications. *Pediatric Nephrology*.

[96] Bernheim J., Benchetrit S. (2011). The potential roles of FGF23 and Klotho in the prognosis of renal and cardiovascular diseases. *Nephrology, Dialysis, Transplantation*.

[97] Hu M. C., Shi M., Cho H. J. (2015). Klotho and phosphate are modulators of pathologic uremic cardiac remodeling. *Journal of the American Society of Nephrology*.

[98] Bergmark B. A., Udell J. A., Morrow D. A. (2019). Klotho, fibroblast growth factor-23, and the renin-angiotensin system – an analysis from the PEACE trial. *European Journal of Heart Failure*.

[99] Seiler S., Cremers B., Rebling N. M. (2011). The phosphatonin fibroblast growth factor 23 links calcium-phosphate metabolism with left-ventricular dysfunction and atrial fibrillation. *European Heart Journal*.

[100] Chua W., Purmah Y., Cardoso V. R. (2019). Data-driven discovery and validation of circulating blood-based biomarkers associated with prevalent atrial fibrillation. *European Heart Journal*.

[101] Kuczera P., Adamczak M., Wiecek A. (2016). Fibroblast growth factor-23-a potential uremic toxin. *Toxins*.

[102] Moe S. M., Chertow G. M., Parfrey P. S. (2015). Cinacalcet, fibroblast growth factor-23, and cardiovascular disease in hemodialysis: the evaluation of cinacalcet HCl therapy to lower cardiovascular events (EVOLVE) trial. *Circulation*.

[103] Yamazaki Y., Tamada T., Kasai N. (2008). Anti-FGF23 neutralizing antibodies show the physiological role and structural features of FGF23. *Journal of Bone and Mineral Research*.

[104] Shalhoub V., Shatzen E. M., Ward S. C. (2012). FGF23 neutralization improves chronic kidney disease-associated hyperparathyroidism yet increases mortality. *The Journal of Clinical Investigation*.

